# Transcriptomic Portraits and Molecular Pathway Activation Features of Adult Spinal Intramedullary Astrocytomas

**DOI:** 10.3389/fonc.2022.837570

**Published:** 2022-03-21

**Authors:** Nikolay Konovalov, Stanislav Timonin, Dmitry Asyutin, Mikhail Raevskiy, Maxim Sorokin, Anton Buzdin, Stanislav Kaprovoy

**Affiliations:** ^1^ Burdenko Neurosurgical Center, Moscow, Russia; ^2^ Omicsway Corp., Walnut, CA, United States; ^3^ Moscow Institute of Physics and Technology, Moscow, Russia; ^4^ Shemyakin-Ovchinnikov Institute of Bioorganic Chemistry, Moscow, Russia; ^5^ I.M. Sechenov First Moscow State Medical University, Moscow, Russia; ^6^ Oncobox Ltd., Moscow, Russia

**Keywords:** spinal intramedullary astrocytoma, glioblastoma, transcriptomic (RNA-Seq), RNA sequencing (RNA-seq), molecular pathway activation, gene expression, spinal cord neoplasms

## Abstract

In this study, we report 31 spinal intramedullary astrocytoma (SIA) RNA sequencing (RNA-seq) profiles for 25 adult patients with documented clinical annotations. To our knowledge, this is the first clinically annotated RNA-seq dataset of spinal astrocytomas derived from the intradural intramedullary compartment. We compared these tumor profiles with the previous healthy central nervous system (CNS) RNA-seq data for spinal cord and brain and identified SIA-specific gene sets and molecular pathways. Our findings suggest a trend for SIA-upregulated pathways governing interactions with the immune cells and downregulated pathways for the neuronal functioning in the context of normal CNS activity. In two patient tumor biosamples, we identified diagnostic *KIAA1549-BRAF* fusion oncogenes, and we also found 16 new SIA-associated fusion transcripts. In addition, we bioinformatically simulated activities of targeted cancer drugs in SIA samples and predicted that several tyrosine kinase inhibitory drugs and thalidomide analogs could be potentially effective as second-line treatment agents to aid in the prevention of SIA recurrence and progression.

## Introduction

Spinal intramedullary astrocytoma (SIA) is a rare subtype of glioma comprising about 2%–4% of all primary central nervous system (CNS) neoplasms and approximately 6%–8% of tumors occurring in the spinal cord. SIAs are mainly observed as low-grade tumors (WHO I and II) (1). Five-year overall survival rate of patients with low-grade SIA is 70%–80% and declines to 14%–28% for grades III–IV (2). However, clinical data on prognostic biomarkers and tumor molecular data associated with treatment outcomes are needed for patients with spinal astrocytoma due to a particularly low frequency of these tumors and lack of successful therapeutic regimens. In addition, diagnosis and treatment of these neoplasms is often challenging given their ambiguous manifestations such as back pain, limb weakness, paresthesia, and bowel and bladder dysfunction (1, 3). Spinal cord tumors are more frequently diagnosed in children (3, 4) but also occur in adults (4).

Surgical resection remains the main primary treatment for intramedullary astrocytomas of the spinal cord (3, 5). In turn, second-line treatments usually include radiation therapy and chemotherapy. It was also reported that the use of adjuvant radiation therapy can result in an increase in the overall survival of patients, especially for the lower-grade tumors (6). However, the optimal regimen for an adjunctive therapy including chemotherapy settings has not yet been precisely determined (1, 3, 5). Treating spinal cord astrocytomas remains problematic to date, and morbidity and mortality depend on various factors. In order to better understand these relevant factors linked with the outcomes of spinal cord astrocytomas, several studies were conducted. Due to the complications associated with clinical diagnosis, indecision about optimal surgical treatment, and second-line treatment failures, most reports on intramedullary astrocytomas represent either small cohort retrospective analyses and case studies or data capturing current changes in treatment options (1, 3, 5). Due to the low incidence of these tumors, prospective clinical investigations are problematic to perform, and alternative chemo- and targeted therapeutic agents and regimens were poorly explored for SIA (7–9).

On the other hand, RNA expression profiles may serve as potent predictors of tumor sensitivity to targeted therapeutics, as shown in clinical investigations for microarray (10) and RNA sequencing (RNA-seq) (11) data. Furthermore, molecular pathway activation levels can be calculated using high-throughput gene expression profiles (12, 13) and translated into next-generation biomarkers (14–16) for algorithmic scoring of cancer drug efficiencies (17, 18). Moreover, aggregation of expression data of single gene products into pathways or signatures results in significantly more robust expression-level biomarkers, as deduced theoretically (19) and shown on real cancer molecular data (20, 21). Thus, RNA-seq profiles can be used for finding effective cancer prognostic or predictive biomarkers and assist in finding better clinical treatment regimens (22–24). However, there is a dearth of clinically annotated molecular profiles of SIA that could be used for such a purpose.

In this study, we report 31 new SIA RNA-seq profiles for 25 patients with documented clinical annotations. As far as we know, the current study presents the first clinically annotated RNA-seq dataset of spinal astrocytomas derived from the intradural intramedullary compartment.

We compared these tumor profiles with the previous healthy CNS RNA-seq data of spinal cord and brain samples (24, 25). We identified differentially expressed gene (DEG) sets in SIA and molecular pathways and analyzed the occurrence of known diagnostic and new fusion transcripts. In addition, we calculated prognostic balanced efficiency scores for known targeted drugs and identified a fraction of them that could be potentially helpful as second-line treatment agents to aid in the prevention of SIA recurrence and progression.

## Results

SIAs were not previously characterized on transcriptome-wide level, and in this study, we aimed to analyze RNA-seq profiles of SIA samples in comparison with healthy brain and spinal cord samples obtained from Genotype-Tissue Expression (GTEx) Portal (25) and Atlas of RNA sequencing profiles for normal human tissues (ANTE) database (24).

### Spinal Intramedullary Astrocytoma Diagnosis and Biosamples

**Figure 1:**
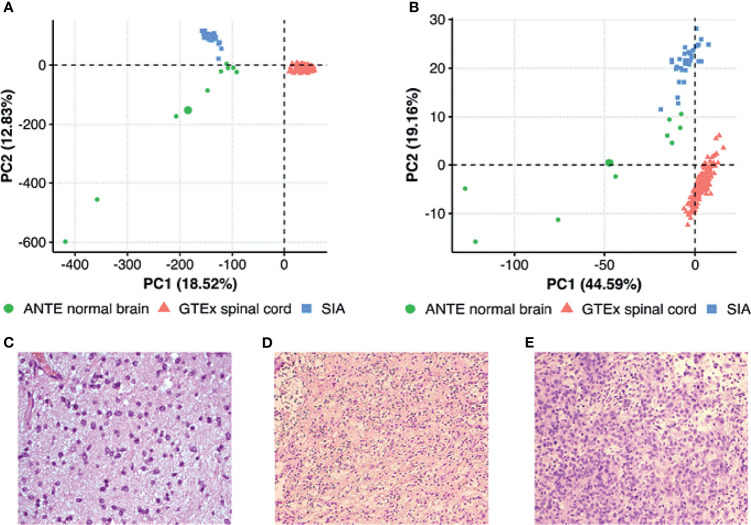
Principal component analysis (PCA) of **(A)** gene expression and **(B)** pathway activation levels (PALs) for spinal intramedullary astrocytoma (SIA) tissue samples and publicly available spinal cord and brain normal samples from The Genotype-Tissue Expression (GTEx) Portal and Atlas of Normal Tissue Expression (ANTE). PALs were calculated according to Borisov et al. (12) with the 168 healthy CNS tissue samples taken as the controls for SIA. Histological examples of **(C)** a diffuse, **(D)** a pilocytic, and **(E)** an anaplastic astrocytoma.

Overall, 31 tumor tissue samples were taken from 14 male and 11 female donors who were diagnosed between 2003 and 2018 with SIA (23 pilocytic astrocytomas, 4 glioblastomas, 2 anaplastic astrocytomas, and 2 astrocytomas with uncategorized histological subtype; **Figure 1**). The mean age was 32.73 years (range 18–69 years) and 30.00 years (18–56 years), respectively. The biosamples were formalin-fixed paraffin-embedded (FFPE) histologically characterized tumor samples with at least 70% cancer cells. Clinical annotations of tumor tissue specimens investigated in this study and their patient origin are summarized in **Supplementary Table S1**. Kaplan–Meier plots for progression-free survival and overall survival are shown in **Supplementary Figure S1**.

### Characteristics of Sequencing and Mapping

A total of 10,509.3 million reads were obtained for 31 independent libraries of SIA tissues (median 55.3 million reads per sample). Most reads reached Phred-like quality scores (Q-scores) at the Q30 level, indicating that the probability of an incorrect base call is 0.001%. The average coverage of sequencing depth reached approximately 53.45× of the human transcriptome. After alignment, 98.57% to 99.12% uniquely aligned reads were mapped to the reference human genome.

### Primary Comparison of RNA Sequencing Profiles of Spinal Intramedullary Astrocytomas and Healthy Central Nervous System Tissues

To further characterize SIA transcriptomic data, we compared using principal component analysis (PCA) distributions of RNA-seq profiles among the SIA samples (n = 31) and publicly available datasets of normal spinal cord (n = 159) and brain (n = 9) tissues from GTEx and ANTE databases, respectively. PCA was performed to investigate cross-dataset compatibility in order to select proper reference group(s) for SIA comparison. The profiles from ANTE database were chosen because they were obtained using the same reagents, equipment, and protocols as for the current experimental SIA sampling (24). The GTEx reference group of samples was selected because this is, to our knowledge, currently the biggest publicly available collection of healthy spinal cord RNA-seq profiles [30]. Performing two-step expression analysis allowed us to select the DEGs between SIA and normal neural tissue explored using the same RNA-seq platform (brain samples from ANTE) and using a different platform but for the same tissue type (GTEx spinal cord samples). Unfortunately, normal spinal cord samples were not available in the ANTE database. We hope that this approach allowed to establish differential gene expression profiles without the influence of platform-specific batch effect.

PCA was performed first in the space of log_10_ transformed quantile normalized gene counts. We observed tissue-specific sample clustering corresponding to the biological nature of the datasets under analysis, where SIA samples formed a separate cluster (**Figure 1** **A**) . In addition, we performed PCA for brain and spinal cord from GTEx, SIA, and ANTE normal brain data normalized using quantile normalization, DESeq2, and harmonized/batch corrected using XPN (26), CuBlock (27), and Shambhala (28). It appeared that GTEx brain profiles clustered with ANTE normal brain even in case of DESeq2 normalization, indicating that further batch correction was not necessary (**Supplementary Figure S2**).

Then, we performed PCA based on pathway activation levels (PALs) of 1,611 molecular pathways (29) calculated using the same transcriptomic data for each sample under study (**Figure 1B**). In case of pathway upregulation or downregulation, PALs can take positive or negative values, respectively, thus quantitatively reflecting the extent of a pathway activation or inhibition relatively to the control group of samples. Zero PAL values suggest unaffected activity of a molecular pathway. Thus, PAL values can be used as the quantitative functional characteristic of the interactome under analysis (14). We calculated PALs according to Borisov et al. (12), with the 168 healthy CNS tissue samples taken as the controls.

On the PAL-based PCA plot, we observed similar clustering as for the gene expression-based PCA (**Figure 1B**), thus strongly suggesting that SIA samples should be independently compared to each of the above healthy CNS tissue datasets.

### Differential Gene Expression Analysis

**Figure 2:**
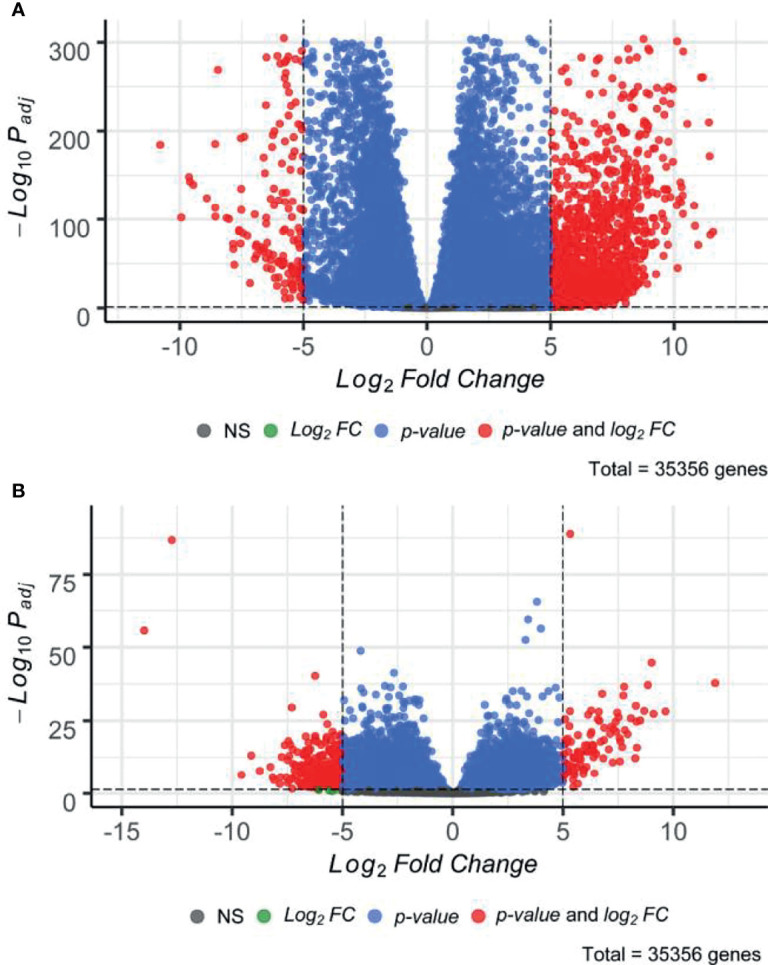
Distribution of differentially expressed genes between spinal intramedullary astrocytomas (SIAs) relative to **(A)** GTEx healthy spinal cord and **(B)** ANTE healthy brain samples.

Subsequently, we performed paired differential gene expression analysis between SIA samples relatively to each dataset of healthy CNS tissues (**Supplementary Table S2**). Overall, 1,949 genes, 1,766 (90.61%) upregulated and 183 (9.39%) downregulated, were found to be statistically significantly differentially expressed [|log_2_ *FC*|>5, false discovery rate (FDR)-adjusted *p*-value <0.05] between SIA and GTEx spinal cord samples (**Figure 2A**). In turn, 382 DEGs, 102 (26.70%) upregulated and 280 (73.30%) downregulated, were found for the comparison between SIA and ANTE healthy brain samples (**Figure 2B**).

**Figure 3:**
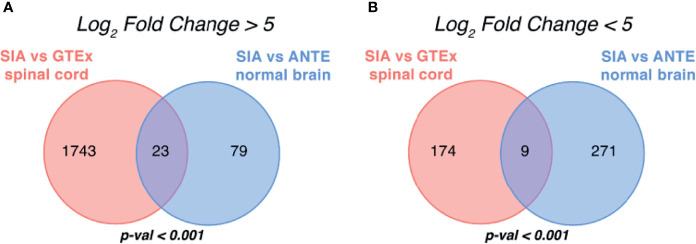
Intersection of differentially expressed gene sets between spinal intramedullary astrocytomas (SIAs), GTEx healthy spinal cord, and ANTE healthy brain samples. Intersections of **(A)** upregulated and **(B)** downregulated differentially expressed gene sets between SIA–GTEx spinal cord and SIA–ANTE normal brain samples are shown; *p*-values for intersection significance obtained in perturbation test are highlighted in bold.

These DEG sets were then intersected with respect to log_2_ *FC* sign (**Figures 3A, B** **)**. In order to test whether an observed number of common differential genes can support random or non-random intersection hypothesis, we performed perturbation test for randomness according to Sorokin et al. (30) with 1,000 random gene sets. The percentile of the observed case precedent in the distribution of random intersections was considered as a measure of statistical significance.

In total, 32 genes, 23 (71.88%) upregulated and 9 (28.12%) downregulated, were commonly differentially expressed in SIA samples according to both comparisons, which supported the hypothesis that the intersections between the DEGs were non-random (*p* < 0.01).

### Gene Ontology Enrichment Analysis

**Figure 4:**
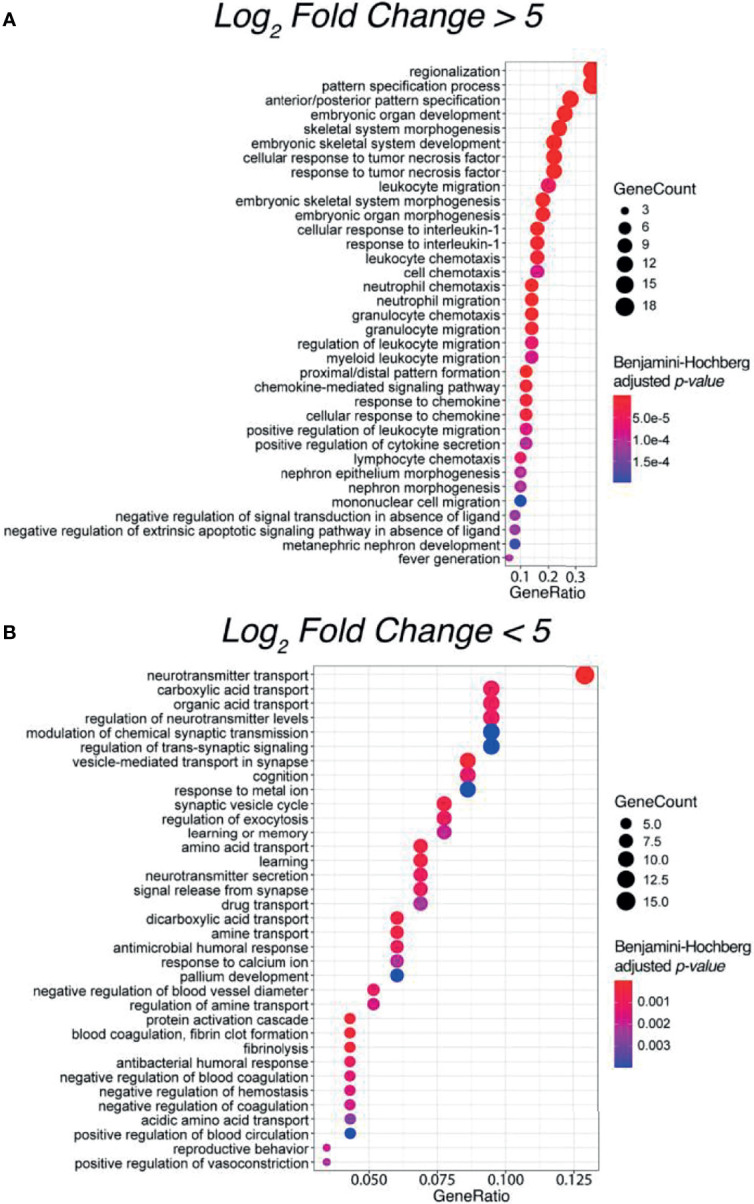
Top 35 enriched Gene Ontology (GO) terms for significantly **(A)** upregulated and **(B)** downregulated differentially expressed genes between spinal intramedullary astrocytomas (SIAs) and healthy CNS tissues: GTEx spinal cord and ANTE normal brain samples.

To evaluate potential functional similarities of the above 32 SIA-specific differential genes and the underlying molecular and cellular processes, we then performed Gene Ontology (GO) terms enrichment analysis (**Figures 4A, B** **)**. We identified significantly enriched 563 functional GO terms, where 340 terms (60.39%) were for upregulated and 223 (39.61%) were for downregulated DEGs. For statistical estimates, we used Benjamini–Hochberg method for FDR correction (31) and *p*-value threshold 0.05 (32).

Interestingly, most of the enriched terms for upregulated DEGs were related to the regulation of an innate and adaptive immune response, thus supporting a concept that immune microenvironment may play a crucial role in the development of spinal astrocytomas (33). In contrast, for downregulated DEGs, the most strongly enriched terms were linked with complex neuronal processes, such as cognition, learning ability, and regulation of neurotransmitter secretion and transport. The latter supports specific functional impairments occurring in astrocytomas in comparison with healthy CNS tissues (34).

### Differential Pathway Activation Analysis

**Figure 5:**
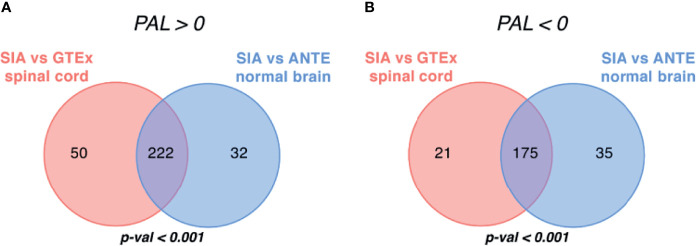
Intersection of differentially regulated molecular pathways between spinal intramedullary astrocytomas (SIAs), GTEx healthy spinal cord, and ANTE healthy brain samples. Intersections of significantly **(A)** upregulated (PAL >0) and **(B)** downregulated (PAL <0) molecular pathways between SIA–GTEx spinal cord and SIA–ANTE normal brain samples are shown; *p*-values for intersection significance obtained in perturbation test are highlighted in bold.

We then performed differential PAL analysis for SIA samples relative to healthy CNS tissues (**Figure 5**). In total, 1,611 molecular pathways including 10 and more gene products were interrogated from Oncobox pathway databank (29).

When comparing SIA and GTEx healthy spinal cord samples, we identified 468 differentially regulated pathways, 272 (58.12%) of them were activated and 196 (41.88%) were suppressed (**Figure 5**). In turn, for the comparison between SIA and ANTE normal brain samples, 464 differential pathways were identified; among them, 254 (54.74%) were activated and 210 (45.26%) were suppressed (**Figure 5**).

The intersections between these pathway sets returned 397 common differential molecular pathways [222 (55.92%) activated and 175 (44.08%) inhibited; **Supplementary Tables S3**, **S4**]. This supported non-random intersection between the differential pathways in both comparisons, *p* < 0.001 (**Figure 5**).

**Figure 6:**
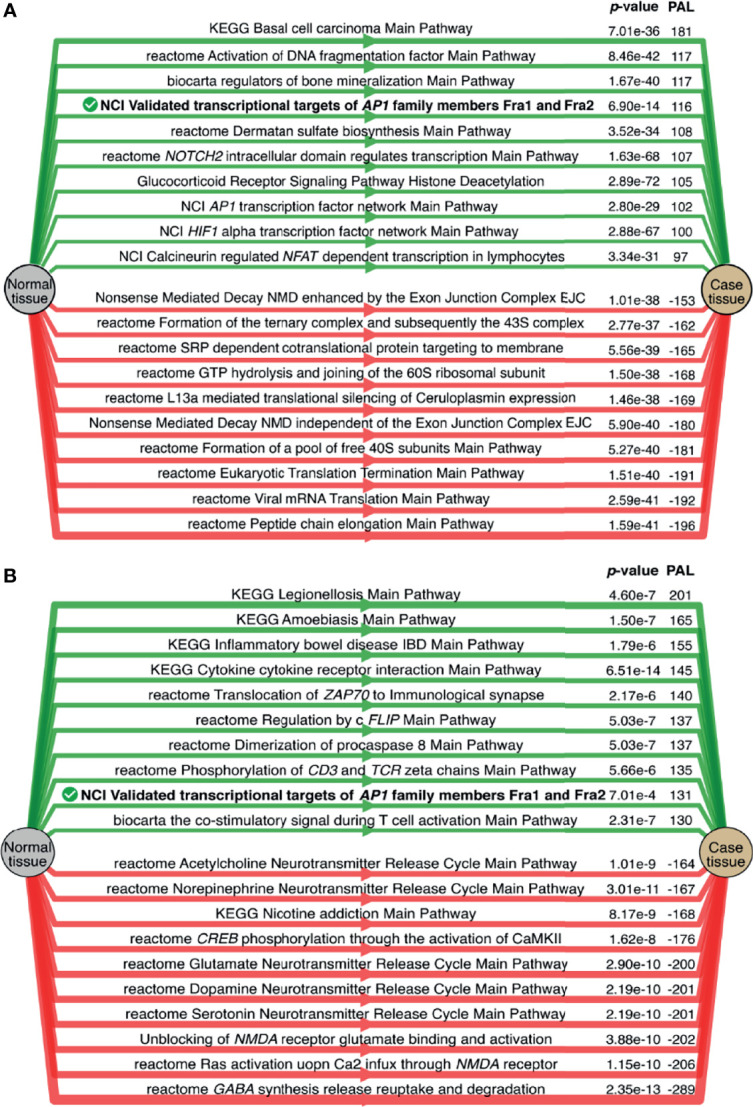
Top 10 activated (green) and suppressed (red) molecular pathways for the comparisons of spinal intramedullary astrocytomas (SIAs) with **(A)** GTEx healthy spinal cord and **(B)** ANTE normal brain samples. Common top differential pathways are shown in bold.

In both comparisons, top activated pathways deal with intracellular signal transduction and with the immune response, whereas top downregulated pathways are responsible for translational regulation and neurotransmitter activities (**Figure 6**). Except for the new feature of translational regulation, this trend was in line with the results obtained previously for the GO terms enrichment in the SIA differential genes (**Figure 4**).

### Simulated Activities of Anticancer Targeted Therapeutics

A chemotherapeutic treatment of SIA remains a challenging and poorly investigated field, and we performed computational simulation whether anticancer targeted drugs (ATDs) that are currently in use for other CNS tumors could be repurposed as second-line treatment options for SIA. To this end, we utilized Oncobox method for predicting efficiencies of ATDs based on gene expression and molecular pathway activation data (35). This returns for every drug a tumor sample-specific value of balanced drug efficiency score [drug score (DS)]. DS reflects an expected responsiveness of a tumor to a specific drug, where higher values mean higher expected efficacy of an ATD. Furthermore, drugs with positive DS are predicted to be potentially beneficial, and drugs with negative DS—potentially harmful (35). This method was shown to be clinically beneficial in a prospective clinical investigation on high-grade human solid tumors [16] and was effective for individual selection of experimental/off-label chemo- and targeted therapeutic settings, e.g., Buzdin et al. (36) and Moisseev et al. (37). In glioblastoma, Oncobox method could effectively predict tumor response on temozolomide, a DNA-alkylating agent whose activity is antagonized by *MGMT* gene products (38).

**Figure 7:**
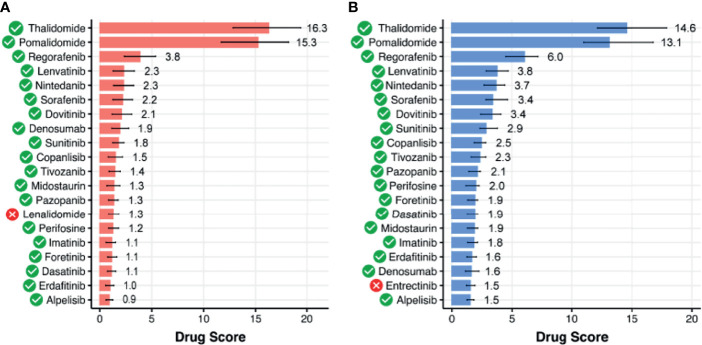
Top 20 targeted therapeutics ranked by drug score for spinal intramedullary astrocytomas (SIAs) separately normalized on **(A)** healthy GTEx spinal cord and **(B)** ANTE normal brain samples. Targeted therapeutics that are common between the two top-20 lists are shown with green marks.

**Figure 8:**
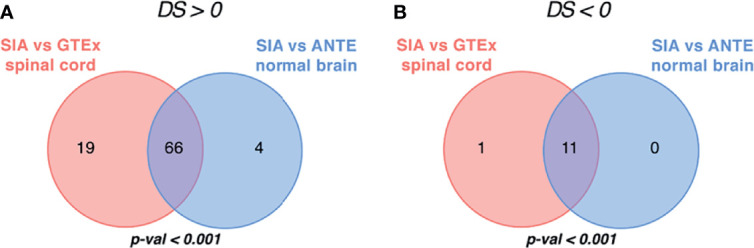
Intersection of targeted therapeutics assessed by Oncobox algorithm with **(A)** positive and **(B)** negative drug score (DS) predicted for spinal intramedullary astrocytomas (SIAs) separately normalized on GTEx healthy spinal cord and on ANTE normal brain samples; *p*-values for intersection significance obtained in perturbation test are highlighted in bold.

By using Oncobox algorithm, we identified 85 ATDs with positive DS in the SIA–GTEx spinal cord comparison, and 70 ATDs with positive DS in the SIA–ANTE healthy brain comparison (**Figures 7**, **8**). In these lists, there were 66 common drugs, thus evidencing non-random intersection between the two comparison results (**Figure 8** and **Supplementary Tables S5**, **S6**).

We then assessed available clinical trial reports for the top 20 DS-ranked drugs among these common 66 ATDs with the biggest DS values for different CNS tumors (**Table 1** and **Figure 7**). Interestingly, the top predicted drugs mostly represented the classes of tyrosine kinase inhibitors (i.e., regorafenib, lenvatinib, nintedanib, sorafenib, dovitinib, sunitinib, tivozanib, pazopanib, imatinib, foretinib, dasatinib, erdafitinib) and thalidomide analogs (thalidomide and pomalidomide). Many of these drugs were previously investigated for CNS tumors and related cancers like neuroblastoma (**Table 1**).

**Table 1:** Overview of existing clinical trials conducted across Central Nervous System (CNS)-related tumors for target drugs with the highest drug score predicted for spinal intramedullary astrocytoma (SIA) samples.

Drug	Nosology	Clinical Trials	Reference
Thalidomide	Advanced secondary glioblastoma	–	(39)
	Neuroblastoma	–	(40)
	Recurrent high-grade gliomas (anaplastic mixed glioma, anaplastic astrocytoma, or glioblastoma multiforme)	Phase II	(41)
	Astrocytoma	Phase II-Thalidomide, Temozolomide Tamoxifen	(42)
Pomalidomide	Pediatric recurrent, progressive, and refractory CNS tumors	Phase I (Phase II failed) NCT02415153	(43)
	Glioblastoma multiforme	–	(44)
Regorafenib	Glioblastoma multiforme	Phase II NCT02926222	(45)
	Recurrent high-grade astrocytoma	- (very poor performance)	(46)
	Neuroblastoma	–	(47)
Lenvatinib	Recurrent glioblastoma multiforme	Phase II NCT01433991	(48)
	Pediatric solid tumors, including CNS tumors	Phase I/II NCT03245151	(49)
Nintedanib (BIBF 1120)	Recurrent glioblastoma multiforme	Phase II (clinically non-relevant antitumor activity) NCT01251484	(50)
	Glioblastomas/Anaplastic oligoastrocytoma/Gliosarcoma/Anaplastic Astrocytoma (AA)/Anaplastic oligodendroglioma (AO)	Phase II NCT01380782	(51)
Sorafenib	Recurrent or progressive low-grade astrocytomas	Phase II NCT01338857	(52)
	Progressive high-grade glioma	–	(53)
	Neuroblastoma	–	(54, 55)
Dovitinib	Recurrent glioblastoma	Phase II NCT01753713	(56)
Sunitinib	Recurrent glioblastoma	Phase II/III NCT03025893	(57)
	Recurrent glioblastoma and anaplastic astrocytoma	Phase II NCT00606008	(58)
	Neuroblastoma	–	(59)
Copanlisib	–	–	–
Tivozanib	Recurrent glioblastoma	Phase II NCT01846871	(60)
Pazopanib	Recurrent glioblastoma	Phase II NCT00459381	(61)
Perifosine	Recurrent glioblastoma	Phase I - Temsirolimus, Perifosine NCT01051557	(62)
	Recurrent or refractory pediatric CNS and solid tumors	Phase I	(63)
Foretenib	Glioblastoma multiforme	–	(64, 65)
Dasatinib	Glioblastoma	- (lack of activity against recurrent glioblastoma)	(66)
Midostaurin	–	–	–
Imatinib	Relapsed/Refractory neuroblastoma	Phase II NCT00030667	(67, 68)
	Recurrent glioblastoma	Phase II NCT00010049	(69)
	Metastatic pilocytic astrocytoma	–	(70)
Denosumab	–	–	–
Alpelisib	–	–	–

On the other hand, drugs with the predicted negative drug scores that were, therefore, algorithmically not recommended belonged mainly to cyclin-dependent kinase inhibitors and androgenic and anabolic steroid (AAS) classes.

### Spinal Intramedullary Astrocytoma Fusion Gene Transcripts

Chromosomal rearrangements resulting in fusion genes and abnormal transcripts in some cases may become clinically actionable targets of specific cancer therapeutics (71). Fusion transcripts combine exons of 2 or more genes and may serve as the oncogenic drivers in many cancers including CNS tumors (72, 73).

**Figure 9:**
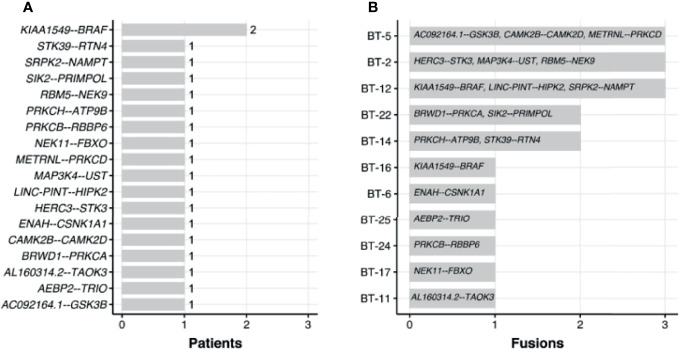
Occurrence of fusions found across spinal intramedullary astrocytoma (SIA) samples by number of patients **(A)** and fusions **(B)**.

**Figure 10:**
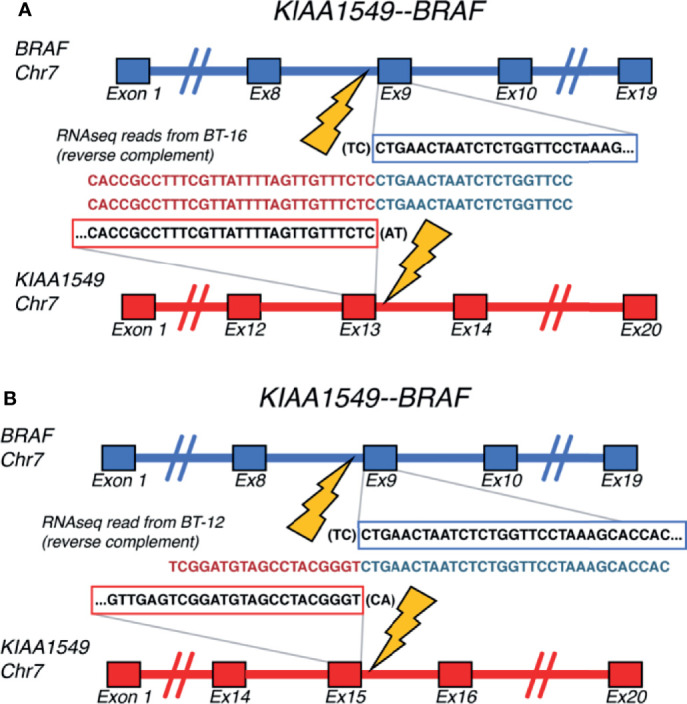
Schematic representation of the *KIAA1549-BRAF* fusion transcripts identified for **(A)** BT-16 and **(B)** BT-12 samples of spinal intramedullary astrocytoma (SIA).

We used RNA-seq profiles for SIA patients to detect fusion transcripts presenting in spinal astrocytoma and focused on the fusions where at least on partner gene was a serine/threonine or tyrosine kinase. This allowed to select potentially druggable fusion genes. We found, in total, 16 different fusion transcripts identified by *STAR-Fusion* software (74) (**Figure 9** and **Supplementary Figure S3**). One of them, *KIAA1549-BRAF*, was found in two SIA patients and preserved BRAF kinase domain (**Figures 10A, B** **)**. Interestingly, this fusion transcript was previously reported to confer a clinically less aggressive phenotype in pediatric low-grade astrocytoma (75). It was also found less abundant in the adult compared to pediatric patients with pilocytic astrocytoma (76). Other fusions, to our knowledge, were not reported previously and were not found in ChimerDB fusion database.

## Discussion

SIAs are rare tumors comprising 6%–8% of all spinal cord tumors, and finding effective lines of treatment for SIA is a challenging task (1, 3). Currently, second-line treatments after surgical resection may include radiation and chemotherapy, where the regimen for adjunctive therapy was not optimally defined. Distance of tumor extension, type of surgery, and adjuvant therapy were significantly associated with SIA patients’ survival in a previous study (77). The limited number of SIA clinical cases results in an absence of prospective studies (7–9). H3-K27 mutation previously showed diagnostic relevance and defined phenotypically and molecularly a distinct set of tumors (78). However, these mutations rarely occur in SIA with just several cases described in the literature (79).

Here, we report the first RNA-seq molecular dataset with documented clinical annotations for 31 samples of 25 SIA patients. While there were studies by Biczok et al. (80) and Zhang et al. (81) on SIA molecular profiling, the current study is, to our knowledge, the only one with publicly available sequencing data. In addition, Biczok et al. (80) performed only targeted RNA-seq for 55 genes to detect fusion genes, while we investigated gene expression profiling using total RNA sequencing.

By comparing the experimental data obtained with the healthy brain and spinal cord CNS tissues, we analyzed SIA-specific DEGs, enrichment of GO terms, activation of molecular pathways, presence of fusion transcripts, and simulated efficacies of anticancer targeted drugs. Our study has certain limitations such as retrospective design and a small number of patients with mixed pathology of low-grade and high-grade spinal cord astrocytomas, although taking into account the rarity of this disease, this is expected.

Our results provide clues on possible molecular mechanisms of spinal astrocytoma and on its biomarkers. Indeed, a group of 23 differential SIA-upregulated genes found in the study was significantly enriched by GO terms mostly linked with regulation of innate and adaptive immune response. This strongly supports a role of the immune microenvironment in SIA development and progression. At the same time, a group of nine differentially downregulated genes was enriched by the terms dealing with neuronal and cognitive functions, thus reflecting their impairment in the cancer tissue.

Interestingly, a secreted extracellular matrix protein periostin was among the 23 SIA upregulated genes. Periostin was previously associated with prognosis and performance status in gliomas (82). Moreover, Mikheev et al. (83) showed that periostin knockdown impaired the survival of xenografted glioma stem cells and thus concluded that targeting periostin may be a promising strategy. Our study supports these findings and points to a potential role of periostin also in spinal astrocytomas.

Furthermore, our algorithmic simulation predicted that 66 targeted therapeutics can be potentially beneficial for SIA treatment. Some of them were already tested for CNS tumors and passed Phases I or II of clinical trials. We speculate that they could be repurposed from being used in other CNS tumors, and related tumors such as neuroblastoma, to improving the second-line treatment of SIA. Interestingly, the most highly ranked drugs (thalidomide and its derivatives) also reflect the top GO terms enriched in the SIA-upregulated gene set, i.e., the cellular response on tumor necrosis factor (**Figure 4A**), whose pathway is a primary molecular target for these drugs (84). Many tyrosine kinase inhibitory drugs were also predicted to be beneficial in SIA treatment.

In contrast, there are some cancer therapeutics we predicted to be potentially harmful for treating SIA, which mainly related to cyclin-dependent kinase inhibitors (*-Ciclibs*) and AAS hormones.

At the level of molecular pathway analysis, we could identify several specific molecular features of SIA. For example, the top upregulated pathways were associated with transcriptional targets of AP1 (Activator Protein-1) family member transcription factors *FOSL1* and *FOSL2.* Interestingly, AP1 transcription factors FOS and Fra1 were found to be upregulated in pilocytic astrocytomas (85). Moreover, Fra1 was shown to control architecture and migratory nature of glioblastoma cells (86). This protein is also linked with promotion of glioma aggressiveness through epithelial–mesenchymal transition (87) and overall glioblastoma invasion (88). In turn, downregulation of Fra1 enhances drug sensitivity in breast cancer cells (89). Also, experimental Fra1 inhibitors significantly suppressed tumor growth and lymph node metastasis of head and neck cancers in a patient-derived xenograft model (90). Thus, our results suggest that Fra1 could be investigated as a potential drug target in rare CNS tumors, such as SIA.

Finally, 16 different fusion transcripts identified in this study suggest the occurrence of chromosomal rearrangements resulting in fusion oncogenes and abnormal transcripts in SIA. Moreover, *KIAA1549-BRAF* fusion detected for two adult SIA patients was previously relatively frequently found in pediatric pilocytic astrocytomas [47] and was reported to confer a clinically less aggressive phenotype in pediatric low-grade astrocytoma (75). Thus, fusion transcripts found can be potentially clinically relevant for SIAs and should be further tested, since many gene fusions were reported as oncogenic drivers in CNS tumors (72, 73).

## Materials and Methods

### Experimental Clinical Biosamples

This study was performed in agreement with the ethical principles of Declaration of Helsinki. Retrospective biosamples were obtained from patients diagnosed with SIAs who had undergone surgery at the spinal department of Burdenko Neurosurgical Center, Moscow. From all the patients involved or from their legal representatives, informed written consents to participate in this study were collected. The study design and consent collection procedure were approved by the local ethical committee of the Burdenko Neurosurgical Center. For all patients enrolled and for their biosamples, the consent was obtained for publication of age, sex, histological tumor type, diagnosis, and molecular data including RNA-seq profiles but not including whole-genome and/or whole-exome sequencing data.

Biosamples were FFPE tumor tissue blocks that were evaluated and confirmed by a pathologist who estimated a proportion of tumor cells and determined the histological type of a tumor. In this study, only FFPE blocks with at least 70% tumor cells were analyzed. In total, 31 samples for 25 SIAs meeting the above criteria were obtained for further molecular screenings (**Supplementary Table S5**).

### Preparation of Libraries and RNA Sequencing

RNA libraries were generated and sequenced according to Suntsova et al. (24). RNA was extracted using RecoverAll™ Total Nucleic Acid Isolation Kit (Invitrogen). RNA concentrations were measured with Qubit RNA Assay Kit, and Agilent 2100 bioanalyzer was used to measure RNA Integrity Number (RIN). Depletion of ribosomal RNA was performed using RNA Hyper Kit (Roche), and then library concentrations and fragment length distributions were measured with Qubit (Life Technologies) and Agilent Tapestation (Agilent), respectively. The RNA-seq was performed using Illumina NextSeq 550 engine for 50-bp single-end reads and approximately 30 million raw reads per sample using standard protocol. Single-end sequencing was used because SIA samples were FFPE tissue blocks that typically have a strong degree of RNA degradation. Primary sequencing data quality control was performed with Illumina SAV, and demultiplexing was made according to Suntsova et al. (24) with Illumina Bcl2fastq2 v 2.17 software.

### RNA Sequencing Data Processing

SIA profiles were processed according to Suntsova et al. (24). STAR aligner (91) was used to process FASTQ files from RNA-seq in “GeneCounts” mode for Ensembl human transcriptome annotation GRCh38.89. The gene names for 36,596 annotated genes were converted to HGNC (HUGO Gene Nomenclature Committee) gene symbols from Ensembl IDs according to Complete HGNC dataset, version of August 17, 2021 (https://www.genenames.org). Further quality control metrics for RNA-seq data were obtained with NCBI MAGIC software (92–94). RNA-seq profiles were preprocessed by quantile normalization method (95), and then differential expression analysis was performed using *DESeq2* (96), and visualized with R package *EnhancedVolcano* (97). Genes that were considered significantly differentially expressed had to pass a threshold of FDR-adjusted *p*-values <0.05 (98). GO enrichment analysis was conducted using *clusterProfile* (v.4.2.1) and *org.Hs.eg.db* (v.3.8.2) R packages. Fusion transcripts were detected with *STAR-Fusion* tool (74), and PCA and visualization were done for log_10_ transformed counts of all genes using *pca2d* R (v.3.6.0) and *prcomp* software. Code for data analysis is available at: https://github.com/raevskymichail/SIA_analysis.

### Healthy Tissue Transcriptomic Data

We used healthy tissue transcriptomic profiles obtained for normal human spinal cord biosamples from GTEx project portal (25) and for normal brain samples from the ANTE database (24). In total, 9 ANTE and 159 GTEx normal CNS samples were analyzed. Raw count quantification in GENCODEv26 annotation was obtained from GTEx portal.

### Calculation of Pathway Activation Levels

Algorithmically annotated molecular pathway graphs were taken from our previously published database (29). PALs were calculated with the Oncobox bioinformatic platform. It allows quantitative assessment of PALs using RNA-seq data and functionally annotated collection of molecular pathways (12, 29). We used a set of 1,611 pathways with 10 or more gene products included because of previously reported poor theoretically estimated data aggregation effect for smaller pathways (15).

This method of calculating PAL showed a strong potential to suppress batch effects (15, 16, 21) and to minimize the artifacts introduced by the methods of experimental transcriptome analysis (13, 99). An absolute value of PAL reflects the strength of the pathway up/downregulation, while a positive or negative sign indicates its activation or suppression, accordingly (12). To calculate PAL, each sample RNA-seq profile was normalized on mean geometric levels of gene expression in the relevant control dataset.

### Targeted Drug Efficiency Simulation

Drug score [Balanced Efficiency Score (BES)] for cancer-targeted drugs was calculated according to Tkachev et al. (35), whose method is based on the analysis of targeted molecular pathway activation and relative expression levels of drug target genes.

### Testing of Intersection Significance

To test whether an observed number of overlapping differential genes or pathways between the two intersecting datasets is significant, for every comparison, we performed 1,000 random intersections according to Sorokin et al. (30). In every case, two random samples from the corresponding gene sets under comparison were taken. Then, these random samples were intersected for 1,000 iterations, and numbers of randomly obtained common genes were registered. Then, *p*-value of intersection significance was calculated as an expected fraction of random intersects that is equal to or higher than the experimentally observed number of overlapping genes.

## Conclusions

We report here the first clinically annotated RNA-seq dataset for 31 tumor tissue samples of 25 patients with SIA, a rare CNS tumor. Bioinformatic analysis revealed the presence of characteristic *KIAA1549-BRAF* fusion transcripts in two samples and 16 new fusions each present in one SIA patient. For the first time, differential gene and molecular pathway analysis showed that the top SIA-upregulated pathways govern interactions with the immune cells, whereas the top inhibited pathways deal with normal neuronal activities. In addition, we found SIA-specific activation of molecular targets for cancer drugs: several tyrosine kinase inhibitors and thalidomide analogs. While this is a theoretical prediction, we propose that they could be further investigated as second-line treatment agents to aid in the prevention of SIA recurrence and progression.

## Data Availability Statement

Transcriptomic data used across the analyses was deposited in the Sequencing Read Archive under BioProject accession: PRJNA763174. Normalized gene counts are presented in **Supplementary Dataset 1**. Description of clinically relevant (age, sex, diagnosis, tumor histotype) information is given in **Supplementary Table S1**.

## Ethics Statement

The studies involving human participants were reviewed and approved by the Ethical Committee of the Burdenko Neurosurgical Center. The patients/participants provided their written informed consent to participate in this study.

## Author Contributions

Software: MR. Visualization: MR. Validation: MR. Writing—original draft: MR, MS, and AB. Writing—review and editing: MR, MS, and AB. Investigation: MR. Data curation: MR. Conceptualization: MS and AB. Methodology: MS. Supervision: MS. Project administration: AB. Patient treatment: NK, DA, SK, and ST. Tissue sample collection: NK, DA, SK, and ST. Writing—review and editing: SK and ST. All authors have read and agreed to the published version of the article.

## Funding

This research was funded by The Russian Foundation for Basic Research, grant number 18-29-01042.

## Conflict of Interest

Author MR was employed by Omicsway Corp and AB was employed by Omicsway Corp. and Oncobox Ltd.

The remaining authors declare that the research was conducted in the absence of any commercial or financial relationships that could be construed as a potential conflict of interest.

## Publisher’s Note

All claims expressed in this article are solely those of the authors and do not necessarily represent those of their affiliated organizations, or those of the publisher, the editors and the reviewers. Any product that may be evaluated in this article, or claim that may be made by its manufacturer, is not guaranteed or endorsed by the publisher.

## Supplementary Material

Supplementary Figure 1 Kaplan-Meier plots for progression-free and overall survival.

Supplementary Figure 2 PCA plots for log-transformed counts processed using **(A)** quantile normalization; **(B)** DESeq2 normalization; **(C)** XPN; **(D)** CuBlock; **(E, F)** Shambhala.

Supplementary Figure 3 Schematic representation of 16 different fusion transcripts identified by STAR-Fusion for spinal intramedullary astocytoma (SIA) transcriptomic samples

Supplementary Data Sheet 1 Normalized gene counts.

Supplementary Table 1 Clinical annotations of spinal intramedullary astrocytomas tissue specimens analysed in this study.

Supplementary Table 2 Gene lists for the intersection of upregulated and downregulated differentially expressed gene sets between spinal intramedullary astrocytomas (SIA), GTEx healthy spinal cord, and ANTE healthy brain samples

Supplementary Table 3 Pathway-activation-levels (PAL) from the differential pathway activation analysis estimated for 1611 molecular pathways from Oncobox pathway databank between spinal intramedullary astrocytomas (SIA) and GTEx healthy spinal cord samples.

Supplementary Table 4 Pathway-activation-levels (PAL) from the differential pathway activation analysis calculated across 1611 molecular pathways from Oncobox pathway databank between spinal intramedullary astrocytomas (SIA) and ANTE normal brain samples.

Supplementary Table 5 Simulated activities of anticancer targeted therapeutics (Drug Scores), calculated between spinal intramedullary astrocytomas (SIA) and GTEx healthy spinal cord samples.

Supplementary Table 6 Simulated activities of anticancer targeted therapeutics (Drugs Scores), estimated between spinal intramedullary astrocytomas (SIA) and ANTE normal brain samples.
