# Supplementary figures and images for: Transcriptomic Portraits and Molecular Pathway Activation Features of Adult Spinal Intramedullary Astrocytomas
Source: Front Oncol. 2022 Mar 21;12:837570. doi: 10.3389/fonc.2022.837570 (PMC8978956; doi:10.3389/fonc.2022.837570)

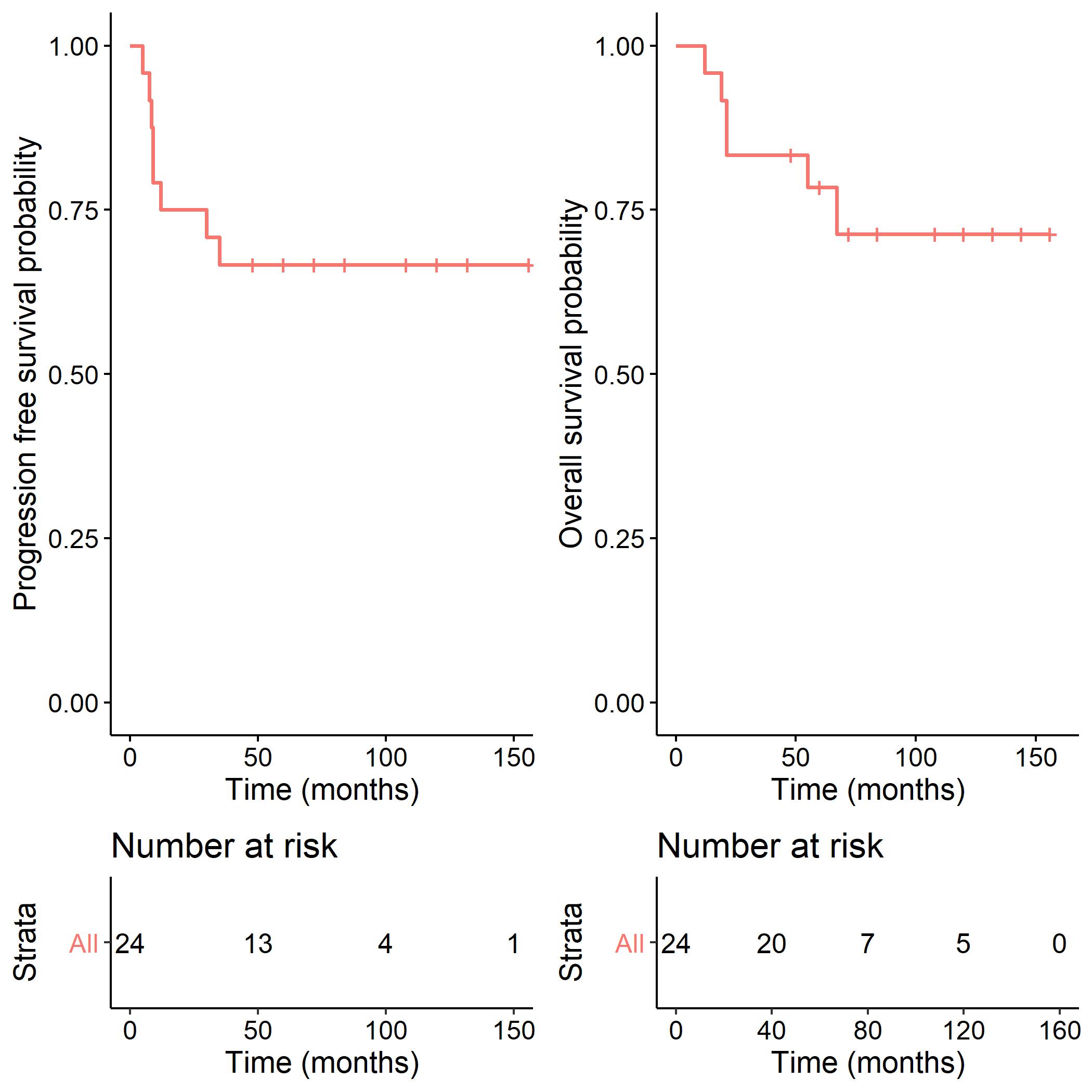

Supplement: Supplementary Figure 1 — Kaplan-Meier plots for progression-free and overall survival. [file DataSheet_1.zip › Figure S1. Surival_SIA.jpeg]

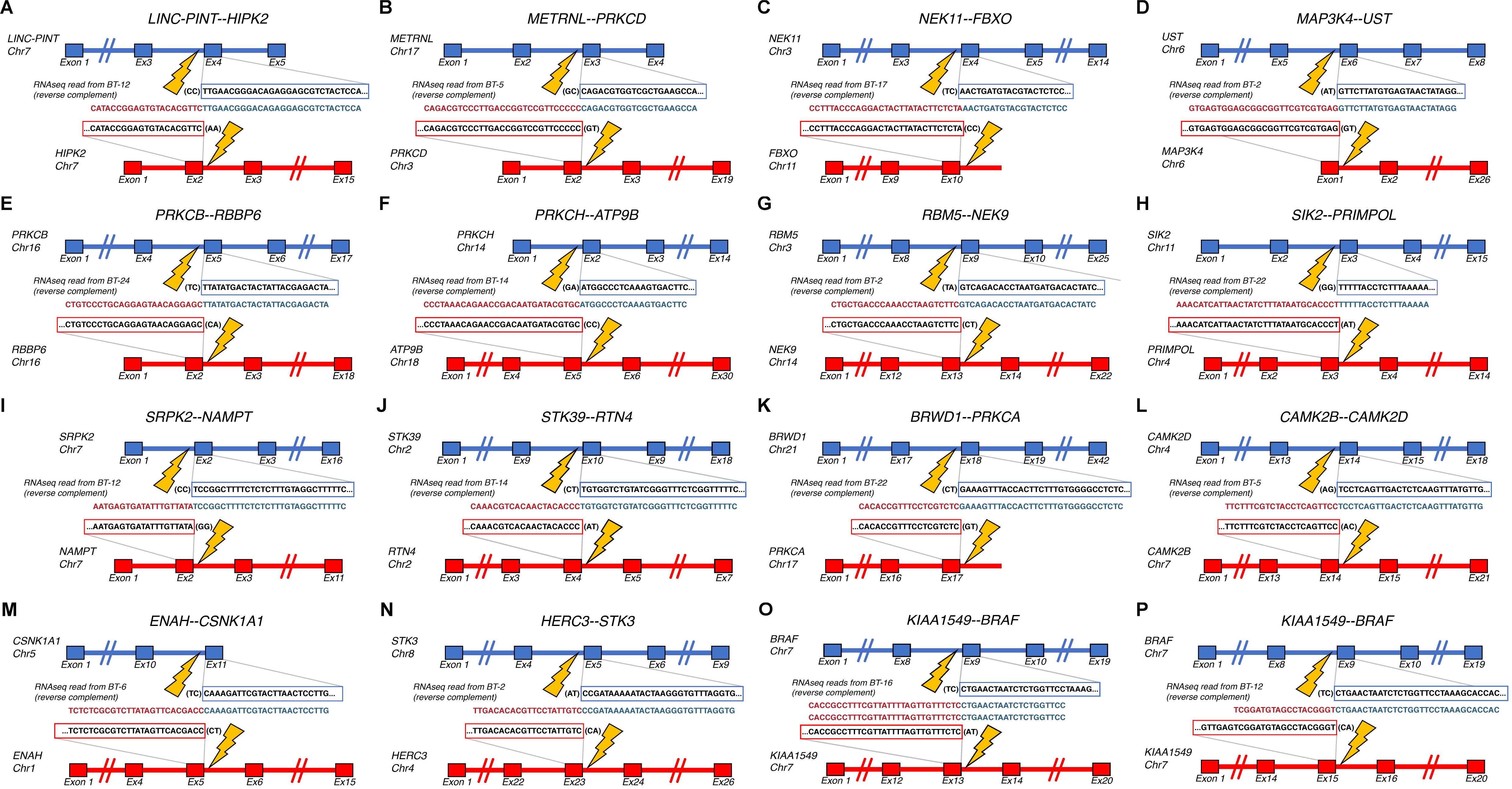

Supplement: Supplementary Figure 1 — Kaplan-Meier plots for progression-free and overall survival. [file DataSheet_1.zip › Figure S3. Fusions.tif]
